# Causal associations of intelligence with schizophrenia and bipolar disorder: A Mendelian randomization analysis

**DOI:** 10.1192/j.eurpsy.2021.2237

**Published:** 2021-10-13

**Authors:** Kazutaka Ohi, Kentaro Takai, Ayumi Kuramitsu, Shunsuke Sugiyama, Midori Soda, Kiyoyuki Kitaichi, Toshiki Shioiri

**Affiliations:** 1 Department of Psychiatry, Gifu University Graduate School of Medicine, Gifu, Japan; 2 Department of General Internal Medicine, Kanazawa Medical University, Ishikawa, Japan; 3 Laboratory of Pharmaceutics, Department of Biomedical Pharmaceutics, Gifu Pharmaceutical University, Gifu, Japan

**Keywords:** Bipolar disorder, GWAS, intelligence, Mendelian randomization, schizophrenia, SNP

## Abstract

**Background:**

Intelligence is inversely associated with schizophrenia (SCZ) and bipolar disorder (BD); it remains unclear whether low intelligence is a cause or consequence. We investigated causal associations of intelligence with SCZ or BD risk and a shared risk between SCZ and BD and SCZ-specific risk.

**Methods:**

To estimate putative causal associations, we performed multi-single nucleotide polymorphism (SNP) Mendelian randomization (MR) using generalized summary-data-based MR (GSMR). Summary-level datasets from five GWASs (intelligence, SCZ vs. control [CON], BD vs. CON, SCZ + BD vs. CON, and SCZ vs. BD; sample sizes of up to 269,867) were utilized.

**Results:**

A strong bidirectional association between risks for SCZ and BD was observed (odds ratio; OR_SCZ → BD_ = 1.47, *p* = 2.89 × 10^−41^, OR_BD → SCZ_ = 1.44, *p* = 1.85 × 10^−52^). Low intelligence was bidirectionally associated with a high risk for SCZ, with a stronger effect of intelligence on SCZ risk (OR_lower intelligence → SCZ_ = 1.62, *p* = 3.23 × 10^−14^) than the reverse (OR_SCZ → lower intelligence_ = 1.06, *p* = 3.70 × 10^−23^). Furthermore, low intelligence affected a shared risk between SCZ and BD (OR _lower intelligence → SCZ + BD_ = 1.23, *p* = 3.41 × 10^−5^) and SCZ-specific risk (OR_lower intelligence → SCZ*vs*BD_ = 1.64, *p* = 9.72 × 10^−10^); the shared risk (OR_SCZ + BD → lower intelligence_ = 1.04, *p* = 3.09 × 10^−14^) but not SCZ-specific risk (OR_SCZ*vs*BD → lower intelligence_ = 1.00, *p* = 0.88) weakly affected low intelligence. Conversely, there was no significant causal association between intelligence and BD risk (*p* > 0.05).

**Conclusions:**

These findings support observational studies showing that patients with SCZ display impairment in premorbid intelligence and intelligence decline. Moreover, a shared factor between SCZ and BD might contribute to impairment in premorbid intelligence and intelligence decline but SCZ-specific factors might be affected by impairment in premorbid intelligence. We suggest that patients with these genetic factors should be categorized as having a cognitive disorder SCZ or BD subtype.

## Introduction

Schizophrenia (SCZ) and bipolar disorder (BD) are common psychiatric disorders with a lifetime morbidity rate of approximately 1% [1,2]. These psychiatric disorders are the leading cause of years lived with disability worldwide [3]. Both disorders are highly heritable with an estimated heritability of approximately 80% [4,5]. To find risk genes for these disorders, large-scale genome-wide association studies (GWASs) for SCZ and BD have been performed by the Psychiatric Genomics Consortium (PGC), identifying 108 and 30 distinct genomic loci related to the risk for SCZ and BD, respectively [6,7]. Substantial overlap between SCZ and BD has been demonstrated with a high genetic correlation (*r_g_* = 0.7–0.8) derived from common genetic variants (SNPs) [7,8]. Despite the shared genetics, the current diagnostic criteria (Diagnostic and Statistical Manual of Mental Disorders, fifth edition [DSM-5]) adhere to the historical distinctions between SCZ and BD since the late 19th century. These disorders were differentiated as independent categorical diagnostic entities based on their clinical presentation with psychotic (positive and negative) symptoms in SCZ and manic symptoms in BD.

Cognitive impairment relatively independent of psychotic and manic symptoms is a core feature of SCZ and BD [9–13], although cognitive impairment is not included in the previous and current diagnostic criteria (DSM-IV and 5) for the disorders. Cognitive impairment is a predictor of poor functional outcomes, such as social and occupational dysfunction [14–16]. Patients with SCZ and BD show impairment in premorbid intelligence as well as in current intelligence, which involves intelligence decline from the premorbid level [9,12,17]. Intelligence is also substantially heritable with an estimated heritability of approximately 50–70% [18,19]. Large-scale GWASs using nearly 300,000 healthy individuals of general population-based cohorts have detected more than 100 genome-wide significant loci related to intelligence [20,21].

The highly heritable disorders SCZ and BD have clinical similarities, such as low intelligence [22–24]. In general, identifying genetic components contributing to these disorders will provide insight into the biology underlying their shared impairments. On the other hand, as SCZ and BD are distinct diagnoses according to DSM-5, SCZ and BD may have disorder-specific genetic factors. To date, the SCZ and BD working groups of the PGC have identified 114 genome-wide significant loci shared between SCZ and BD (SCZ + BD) as well as two genome-wide significant loci differentiating SCZ from BD (SCZ vs. BD) [25]. Although the disorders are associated with impairments in intelligence and there is a high genetic correlation between them, intelligence genetically correlates negatively only with risk for SCZ (*r_g_* = 0.2) and not with risk for BD [26,27]. Current SCZ diagnosis is considered an aggregation of at least two disorder subtypes: one part is a cognitive disorder that is independent of BD, and the other part resembles high intelligence and BD [28]. It is unclear whether intelligence correlates genetically with a shared genetic factor between SCZ and BD and a disorder-specific genetic factor. We hypothesized that intelligence correlates genetically with both shared genetic factors and SCZ-specific genetic factors.

Several risk factors (exposures), such as low intelligence, are associated with common psychiatric disorders (outcomes), such as SCZ and BD. However, these associations are usually derived from observational studies that cannot distinguish whether the risk factors are “upstream” causal factors, “downstream” consequences of the disorders or confounding factors associated with both exposures and outcomes [29]. Randomized controlled trials (RCTs) are the gold standard approach to assess causality from observational epidemiology, yet RCTs are time-consuming, expensive, or sometimes impractical (e.g., no intervention may exist). As SNPs are present from birth and are unlikely to be confounded by environmental factors under the assumption that there is no other confounding factors, for example, population stratification and assortative mating, methods using SNPs are useful to infer causality. Mendelian randomization (MR) is a method that uses SNPs as instrumental variables to test for causative association between an exposure and an outcome [29,30]. There are three key assumptions that must hold for a MR study to be valid: (a) relevance assumption (the SNPs associate with the risk factor of interest), (b) independence assumption (there are no unmeasured confounders of the associations between SNPs and outcome), and (c) exclusion restriction assumption (the SNPs affect the outcome only through their effect on the risk factor of interest) [31]. To date, the unidirectional MR analysis found that lower intelligence increases the likelihood of SCZ [32]. However, causal associations among intelligence, SCZ and BD are still unclear. The current study focused on questions about causality: Does a low level of intelligence cause SCZ or BD? Does SCZ or BD cause intelligence decline? Uncovering the nature of these associations would inform interventional strategies.

In this study, we performed a multi-SNP MR analysis (generalized summary-data-based MR; GSMR) to examine potential causal associations of intelligence with risks for SCZ and BD as well as a shared risk between SCZ and BD (SCZ + BD) and SCZ-specific risk (BD vs. SCZ). We used publicly available summary-level datasets from five GWASs ([a] intelligence, [b] SCZ vs. control (CON), [c] BD vs. CON, [d] SCZ + BD vs. CON, and [d] SCZ vs. BD) to investigate putative causal associations among intelligence, SCZ and BD.

## Methods

### GWAS samples

Five publicly available GWAS summary datasets (intelligence [*n* = 269,867] [20], SCZ [*n* = 33,426] vs. CON [*n* = 32,541] [25], BD [*n* = 20,129] vs. CON [*n* = 21,524] [25], SCZ + BD [*n* = 53,555] vs. CON [*n* = 54,065] [25], and SCZ [*n* = 23,585] vs. BD [*n* = 15,270] [25]) were utilized through the complex trait genetics (CTG) lab (https://ctg.cncr.nl/software/summary_statistics) and PGC (https://www.med.unc.edu/pgc/results-and-downloads) as MR GWAS samples to identify risk SNPs for each phenotype, the frequency of the effect allele, the effect size (beta or odds ratio [OR]), the standard error, the *p* value and the sample size. We selected these GWAS summary statistics to avoid any overlapping samples for our analysis because independent GWASs for SCZ versus CON and BD versus CON have been used to identify disorder-specific genetic variants [25]. The sample information and details regarding the sample collection, genotyping, processing, quality control, and imputation procedures applied in each GWAS have been described previously [20,25].

### Mendelian randomization

To estimate credible causal associations among intelligence, SCZ and BD, we performed MR analyses using the GSMR method (https://cnsgenomics.com/software/gcta/#Mendelianrandomisation) [29] in the genome-wide CTA (GCTA) software v1.93.2beta. The GSMR method examines putative causal associations (*b_xy_* = *b_zx_*/*b_zy_*) between a risk factor (*b_zx_*) and a disorder (*b_zy_*) using summary-level data from GWASs, where *z* is a genotype of a SNP (coded as 0, 1, or 2), *x* is the exposure (e.g., intelligence) in standard deviation (SD) units, and *y* is the outcome (e.g., the liability of a disorder) on the logit scale. *b_zy_* is the effect of *z* on *y* on the logit scale (logarithm of odds ratio, logOR), *b_zx_* is the effect of *z* on *x*, and *b_xy_* is the effect of *x* on *y* free of confounding from nongenetic factors. Near-independent GWAS SNPs (*r^2^* threshold = 0.05, window size = 1 Mb and *p* value threshold = 5.0 × 10^−8^) for each phenotype using ABCD Research Consortium data [33,34] (*n* = 4,920 unrelated individuals of European ancestry) as the reference for linkage disequilibrium (LD) estimation were selected after applying the clumping algorithm in PLINK. First, we estimated putative causal associations (*b_xy_*) of SCZ GWAS SNPs (*p* < 5.0 × 10^−8^) (*b_zx_*) on BD (*b_zy_*) and vice versa in independent samples. Second, putative causal associations (*b_xy_*) of intelligence GWAS SNPs (*p* < 5.0 × 10^−8^) (*b_zx_*) on SCZ or BD (*b_zy_*) were estimated using independent samples, and reverse putative causal associations (*b_xy_*) of GWAS SNPs of SCZ or BD (*b_zx_*) on intelligence (*b_zy_*) were estimated as well. When the phenotype had fewer than 10 independent lead SNPs at the stringent GWAS threshold (*p* < 5.0 × 10^−8^), the threshold was relaxed from *p* < 5.0 × 10^−8^ to *p* < 1.0 × 10^−5^ to obtain a sufficient number of SNPs.

We performed analyses in two ways to test for bidirectionality. First, forward and reverse GSMRs were performed using GWAS SNPs from SCZ and BD as the exposure and outcome variables, respectively. Second, forward GSMR was performed using GWAS SNPs associated with intelligence as the exposure variable and (a) SCZ, (b) BD, (c) a shared factor between SCZ and BD, and (d) a factor differentiating SCZ from BD as the outcomes. In contrast, reverse GSMR was performed using GWAS SNPs from (a) SCZ, (b) BD, (c) a shared factor between SCZ and BD, and (d) a factor differentiating SCZ from BD as exposure variables and intelligence as the outcome. For (b) BD and (d), a factor differentiating SCZ from BD, a threshold of *p* < 1.0 × 10^−5^ was used for the selection of lead SNPs because there were < 10 lead SNPs available at the stringent GWAS threshold (*p* < 5.0 × 10^−8^). To remove horizontal pleiotropic SNPs for both risk factors and disorders, heterogeneity in dependent instrument (HEIDI)-outlier filtering was applied before the analysis, with the default setting (threshold 0.01) [29]. The HEIDI outlier removal strategy to detect SNPs with a horizontal pleiotropic effect is implemented in the GSMR approach that is utilized in this study. The HEIDI attempts to reduce heterogeneity by removing SNPs that contribute to the heterogeneity disproportionately more than expected given the standard errors of the Wald ratios. To generate the effect size plot, we used R v3.6.1 (http://www.r-project.org/) and an R script (gsmr_plot.r) (https://cnsgenomics.com/software/gcta/#Mendelianrandomisation). A Bonferroni-corrected significance threshold of two-tailed *p* < 0.0125 (*α* = 0.05/4 exposure variables) was used to avoid type I error.

## Results

### Effects of risk for SCZ on risk for BD, and vice versa

**Figure 1.**
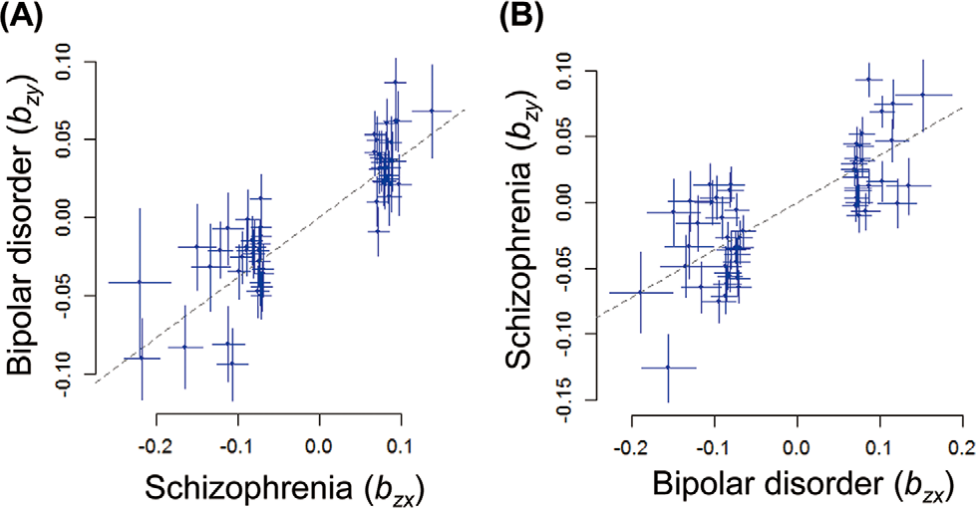
A bidirectional effect (*b_xy_*) of risk for SCZ (*b_zx_*) on risk for BD (*b_zy_*) (a) and of risk for BD (*b_zx_*) on risk for SCZ (*b_zy_*) (b). BD, bipolar disorder; SCZ, schizophrenia. We plotted effect sizes of independent lead SNPs from the GWAS of *b_zx_* on the *x*-axis and SNP GWAS effect sizes for *b_zy_* on the *y*-axis. The dotted line shows a line with a slope of *b_xy_* and an intercept of 0. Error bars represent 95% confidence intervals for the effect sizes for each disorder.

We first investigated a bidirectional effect of risk for SCZ on risk for BD and of risk for BD on risk for SCZ by MR (Figure 1 and Table 1). As expected, we found a strong bidirectional effect of risk for SCZ on risk for BD (OR_SCZ → BD_ = 1.47, *b_xy_* = 0.387, *p* = 2.89 × 10^−41^) and of risk for BD on risk for SCZ (OR_BD → SCZ_ = 1.44, *b_xy_* = 0.362, *p* = 1.85 × 10^−52^). Both directions of the causation were highly significant, and the degrees of effect sizes were similar. Both risks for these psychotic disorders were associated with each other.

**Table 1.** Bidirectional causal associations between disorders and between lower intelligence and risks for SCZ, BD, SCZ + BD, or SCZ versus BD.

Phenotypes		GSMR results				
Exposure (*b_zx_*)	Outcome (*b_zy_*)	*b_xy_*	SE	OR	*p* value	index SNPs (*N*)
SCZ	BD	0.387	0.029	1.47	**2.89 × 10**^**−41**^	55
BD	SCZ	0.362	0.024	1.44	**1.85 × 10**^**−52**^	57
lower intelligence	SCZ	0.482	0.063	1.62	**3.23 × 10**^**−14**^	116
SCZ	lower intelligence	0.056	0.006	1.06	**3.70 × 10**^**−23**^	48
lower intelligence	BD	0.083	0.075	1.09	0.27	125
BD	lower intelligence	−0.009	0.005	0.99	0.06	52
lower intelligence	SCZ + BD	0.207	0.050	1.23	**3.41 × 10**^**−5**^	113
SCZ + BD	lower intelligence	0.042	0.006	1.04	**3.09 × 10**^**−14**^	75
lower intelligence	SCZ vs. BD	0.493	0.081	1.64	**9.72 × 10**^**−10**^	129
SCZ vs. BD	lower intelligence	−0.001	0.008	1.00	0.88	19

Abbreviations: BD, bipolar disorder; GSMR, generalized summary-data-based Mendelian randomization; SCZ, schizophrenia; SE, standard error; OR, odds ratio. *P* values are shown in boldface if *p* < 0.05.

### Effects of lower intelligence on risk levels of SCZ or BD, and vice versa

**Figure 2.**
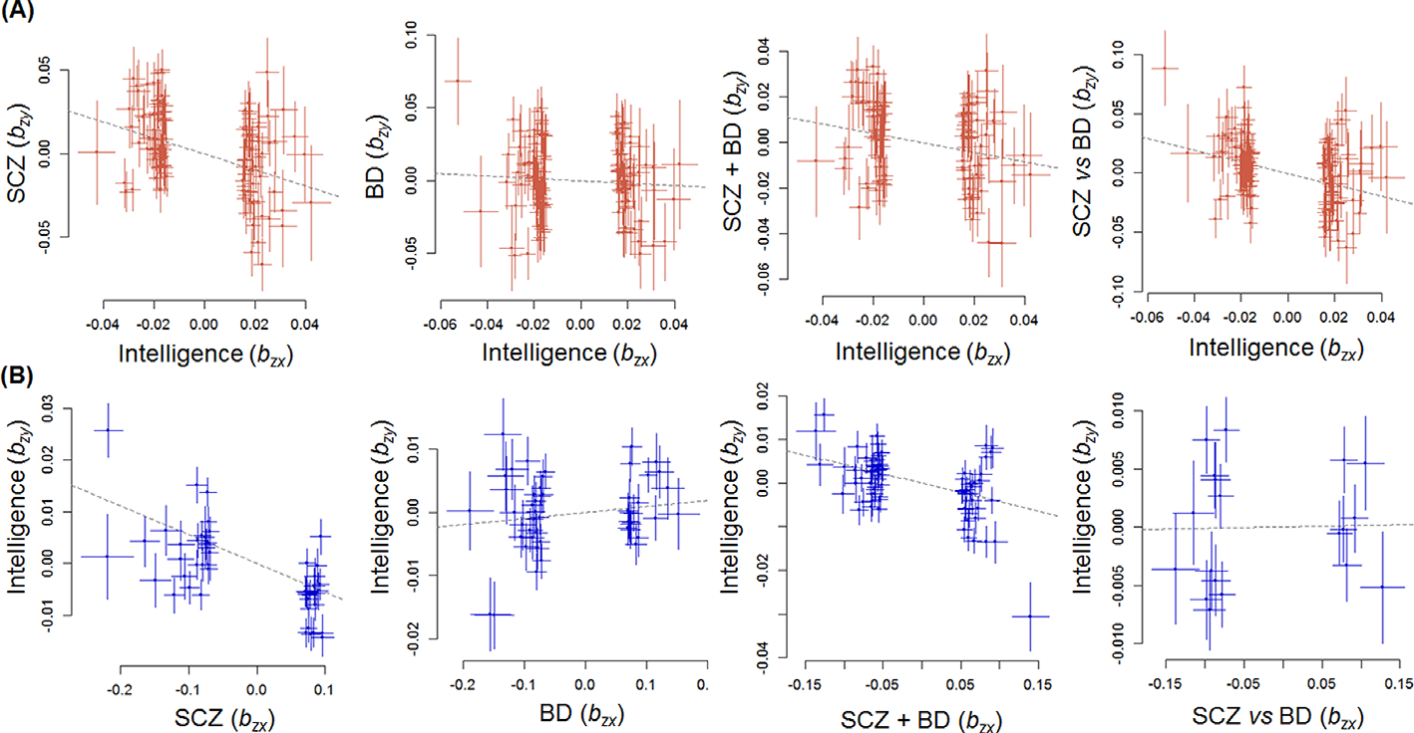
A bidirectional effect (*b_xy_*) of intelligence (*b_zx_*) on risk for SCZ or BD, a shared risk between SCZ and BD (SCZ + BD), or SCZ-specific risk (SCZ vs. BD) (*b_zy_*) (a). A bidirectional effect (*b_xy_*) of risk for SCZ or BD, SCZ + BD, or SCZ versus BD (*b_zx_*) on intelligence (*b_zy_*) (b). BD, bipolar disorder; SCZ, schizophrenia.

Next, we investigated causal associations between lower intelligence and risks for SCZ or BD (Figure 2a,b and Table 1) and observed a strong bidirectional effect of lower intelligence on the risk for SCZ (OR_lower intelligence→SCZ_ = 1.62, *b_xy_* = 0.482, *p* = 3.23 × 10^−14^) and of the risk for SCZ on lower intelligence (OR_SCZ → lower intelligence_ = 1.06, *b_xy_* = 0.056, *p* = 3.70 × 10^−23^) with a stronger effect of lower intelligence on the risk for SCZ than the reverse. Lower intelligence was strongly associated with a higher risk for SCZ, whereas a higher risk for SCZ was weakly associated with lower intelligence. In contrast, there were no significant effects of lower intelligence on the risk of BD (OR_lower intelligence→BD_ = 1.09, *b_xy_* = 0.083, *p* = 0.27) or of BD on lower intelligence (OR_BD → lower intelligence_ = 0.99, *b_xy_* = −0.009, *p* = 0.065).

### Effects of lower intelligence on a shared risk between SCZ and BD or SCZ-specific risk, and vice versa

We further tested for bidirectional causal associations between lower intelligence and a shared risk between SCZ and BD (SCZ + BD vs. CON) or a factor differentiating SCZ from BD (SCZ vs. BD), that is, SCZ-specific risk (Figure 2a,b and Table 1). Lower intelligence was associated with higher shared risk between SCZ and BD (OR_lower intelligence→SCZ + BD_ = 1.23, *b_xy_* = 0.207, *p* = 3.41 × 10^−5^) and SCZ-specific risk (OR_lower intelligence → SCZ*vs*BD_ = 1.64, *b_xy_* = 0.493, *p* = 9.72 × 10^−10^). Conversely, a shared risk between SCZ and BD was weakly associated with lower intelligence (OR_SCZ + BD → lower intelligence_ = 1.04, *b_xy_* = 0.042, *p* = 3.09 × 10^−14^), but there was no significant effect of SCZ-specific risk on lower intelligence (OR_SCZ*vs*BD → lower intelligence_ = 1.00, *b_xy_* = −0.001, *p* = 0.88).

## Discussion

We, for the first, time investigated causal associations of intelligence with risks for SCZ or BD as well as a shared risk between SCZ and BD and SCZ-specific risk using MR analyses. As expected, risks for SCZ and BD were bidirectionally causally associated with each other, with similar effect sizes: a higher risk for SCZ causes a higher risk for BD, whereas a higher risk for BD increases the risk for SCZ. Furthermore, we found a bidirectional causal association between intelligence and risk for SCZ but not for BD. Lower intelligence was strongly related to risk for SCZ, yet risk for SCZ was only weakly related to lower intelligence. Consistent with the association between intelligence and SCZ, lower intelligence was strongly related to a shared risk between SCZ and BD; the shared risk was weakly related to lower intelligence. Intriguingly, we revealed a unidirectional causal association between intelligence and SCZ-specific risk, whereby lower intelligence was unidirectionally related to higher risk.

We identified a bidirectional causal association between intelligence and SCZ: a protective effect of higher intelligence against risk for SCZ and a harmful effect of SCZ on intelligence. Overall, the impact of lower intelligence on the risk for SCZ (OR_lower intelligence→SCZ_ = 1.62) was stronger than that of the risk for SCZ on lower intelligence (OR_SCZ → lower intelligence_ = 1.06). The OR of 1.62 can be interpreted as follows: individuals whose intelligence scores are 1 SD below the population mean have 1.62-fold higher risk for SCZ compared with the population prevalence. The OR of 1.06 can be interpreted as patients with SCZ compared with general population mean have 1.06-fold higher risk of having intelligence scores 1 SD below the those of the population. The impact of lower intelligence on risk for SCZ would suggest that lower premorbid intelligence causes SCZ onset and that intelligence, as one of the intermediate phenotypes, mediates the influence of genetic risk on SCZ; however, the impact of risk for SCZ on lower intelligence would imply intelligence decline around the onset of SCZ. These causative associations support observational studies in which patients with SCZ show impairment in premorbid intelligence before the onset of the disorder and the intelligence decline from the premorbid level becomes evident around the time of onset [9,12].

Despite no causative association between intelligence and risk for BD, a bidirectional causative association of intelligence with a shared risk between SCZ and BD was identified. Consistent with the causative association between intelligence and risk for SCZ, the bidirectional effect was observed with a stronger one for intelligence on the shared risk between SCZ and BD (OR_lower intelligence → SCZ + BD_ = 1.23) than the reverse (OR_SCZ + BD → lower intelligence_ = 1.04), supporting observational epidemiology that lower intelligence increases risks of SCZ and BD onset [9,12,17]. Furthermore, higher SCZ + BD polygenic risk scores (PRSs) are associated with more severe illness, such as psychotic symptoms and a greater number of hospitalizations [25] although the study did not examine association between intelligence and the PRSs. In particular, the shared genetic loci implicate neuronal and synaptic pathways shared between the disorders [25]. These findings suggest that the common risk between SCZ and BD is associated with impairment in premorbid intelligence and intelligence decline via neuronal and synaptic dysfunctions and that SCZ or BD patients with shared genetic risk should be categorized as having a cognitive disorder and symptomatic severity subtype in SCZ and BD.

Lower intelligence was unidirectionally related to a higher factor differentiating SCZ from BD, that is, SCZ-specific risk (OR_lower intelligence→SCZ*vs*BD_ = 1.64), suggesting that the SCZ-specific risk is more strongly associated with impairment in premorbid intelligence compared with intelligence decline. Two genome-wide significant loci differentiating SCZ from BD are *DARS2* (aspartyl-tRNA synthetase 2, mitochondrial) and *CSE1L* (chromosome segregation 1 like). DARS2 is suggested to act as a potential molecular marker of early life stress and vulnerability to psychiatric disorders, and CSE1L plays a role in cellular proliferation and apoptosis [25]. Furthermore, *CSE1L* is a potential target gene of miR-137 at SCZ risk loci [6]. These findings suggest that the factors differentiating SCZ and BD are related to impairment in premorbid intelligence through dysregulation of dopaminergic circuits, synaptic plasticity, and myelination during the developmental stage.

Despite the relationship between decreased intelligence and risk for BD in observational studies [9,17], our MR analysis suggests that these traits are not causally related. In the MR analysis, we used GWAS summary statistics based on BD (*n* = 20,129) versus CON (*n* = 21,524) [25] to avoid overlapping CON samples among GWASs, for example, BD versus CON and SCZ versus CON. In contrast, intelligence may be causally related to risks for bipolar I disorder (BD I) or bipolar II disorder (BD II). Therefore, we further explored causal associations between lower intelligence and risks for BD I and BD II using the other GWAS summary datasets based on BD I (*n* = 14,879) versus CON (*n* = 30,992) and BD II (*n* = 3,421) versus CON (*n* = 22,155) [7] (Supplementary Figure S1). Unexpectedly, a higher risk for BD I was weakly associated with higher intelligence (OR_BD I → lower intelligence_ = 0.98, *b_xy_* = −0.025, *p* = 4.41 × 10^−8^). Nonetheless, there were no significant effects of intelligence on the risks of BD I and BD II or of the risk of BD II on intelligence (*p* > 0.05). The population-level correlation between impaired intelligence and risk for BD might be driven by some unobserved confounding factors, such as educational level [32].

There are some limitations to the interpretations of our findings. There are other MR methods other than GSMR, and there are several outlier removal methods that have been used in MR; MR-Egger, Steiger filtering and HEIDI although the detailed concepts were differed among them [35]. The current study applied the HEIDI outlier removal strategy to detect SNPs with a horizontal pleiotropic effect implemented in the GSMR. However, it is necessary to consider the most appropriate method in future studies. There are mainly three assumptions; (a) relevance assumption, (b) independence assumption, and (c) exclusion restriction assumption made in MR [31], and our putative causal relationships should be treated with caution. Weakly associated genetic variants were used in a few our MR analyses. We included nonoverlapping samples of SCZ, BD, and CON, while a part of samples of the CON and nonpsychiatric participants in GWAS for intelligence might be overlapped. The partial overlapping samples would affect our results of the study. The statistical power to estimate *b_xy_* in MR analysis can be greatly improved if *b_zx_* and *b_zy_* are estimated from independent studies using larger sample sizes [36]. Compared with the GWAS sample sizes of intelligence, SCZ versus CON and SCZ + BD versus CON, those of BD versus CON and SCZ versus BD were relatively small, potentially resulting in false positive and negative findings. Genetic variants have a direct effect on the causal trait and an indirect effect on the caused trait. That is, genetic variants are assumed to have no influence on confounding factors that influence both causal and caused traits, and affect the caused trait only through their effect on the causal trait. However, it would be difficult to know a priori whether the assumptions are adequate because recent large-scale genetic studies have performed in samples of mainly of European ancestry (confounding factors) and shown that genetic variants often have effects on several traits (horizontal pleiotropy). Therefore, our findings might be biased by a few violating assumptions.

In conclusion, we demonstrate a bidirectional causal association between intelligence and the risk for SCZ but not the risk for BD using MR analyses. The bidirectional causal association was observed with a stronger effect of intelligence on risk for SCZ than the effect of risk for SCZ on intelligence. These findings support observational studies showing that patients with SCZ display impairment in premorbid intelligence and a decline in intelligence around the onset of the disorder. Furthermore, we found that a shared factor between SCZ and BD might contribute to impairment in premorbid intelligence and intelligence decline but that SCZ-specific factors might be affected by impairment in premorbid intelligence. We suggest that SCZ or BD patients with these genetic factors should be categorized as having a cognitive disorder subtype in SCZ and BD. Future personalized studies using these genetic factors to diagnose and treat SCZ or BD patients are required.

## Data Availability

The summary-level GWAS data from CTG and PGC are available at https://ctg.cncr.nl/software/summary_statistics and https://www.med.unc.edu/pgc/results-and-downloads. The software tools are available at the URLs above.

## Supplementary Materials

To view supplementary material for this article, please visit http://dx.doi.org/10.1192/j.eurpsy.2021.2237. click here to view supplementary material
