# Supplementary material for: Causal associations of intelligence with schizophrenia and bipolar disorder: A Mendelian randomization analysis
Source: Eur Psychiatry. 2021 Oct 13;64(1):e61. doi: 10.1192/j.eurpsy.2021.2237 (PMC8516746; doi:10.1192/j.eurpsy.2021.2237)
Supplement: Supplementary file 1 [file S0924933821022379sup001.doc]

**Supplementary Information**


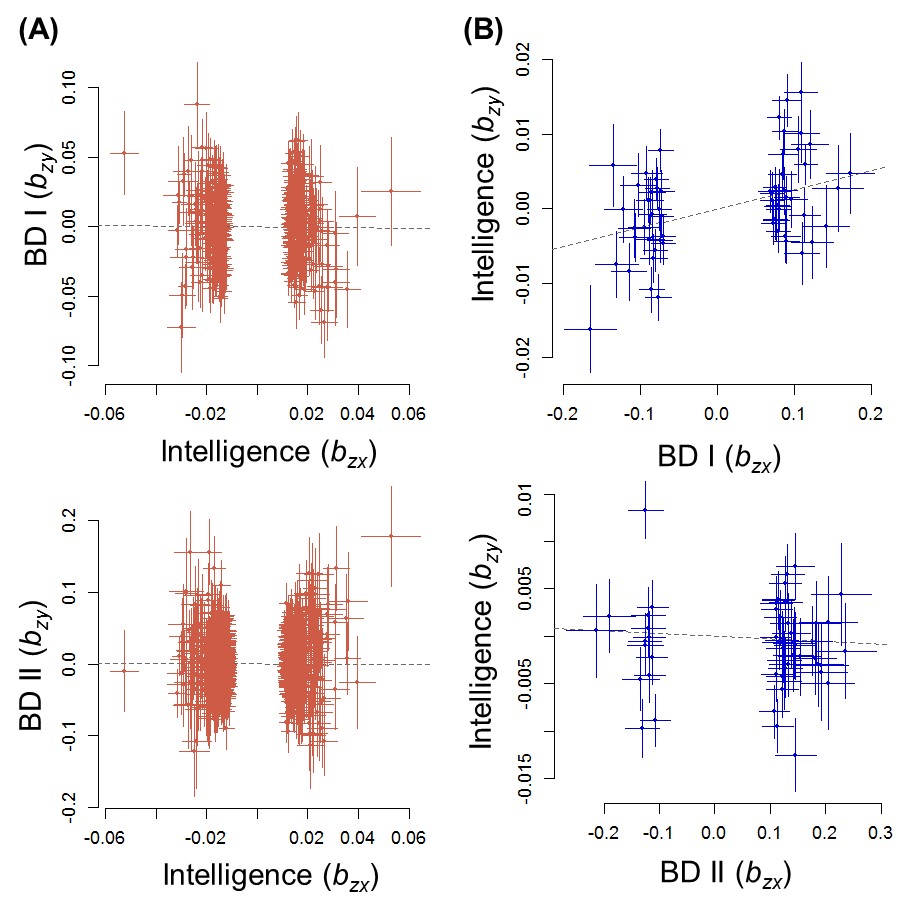


**Supplementary Figure 1.** Effects (*b_xy_*) of intelligence (*b_zx_*) on risk for BD I or BD II (*b_zy_*) **(A)** and risk for BD I or BD II (*b_zx_*) on intelligence (*b_zy_*) **(B)**. Thresholds of *p*<1.0×10^-5^ for BD I *vs* CON and *p*<1.0×10^-4^ for BD II *vs* CON were used for the selection of lead SNPs because there were <10 lead SNPs available at the stringent GWAS threshold (*p*<5.0×10^-8^). BD, bipolar disorder; CON, control.
